# Phenotypic and Genotypic Features of Thai Patients With Nonsyndromic Tooth Agenesis and *WNT10A* Variants

**DOI:** 10.3389/fphys.2020.573214

**Published:** 2020-11-19

**Authors:** Charinya Kanchanasevee, Kanokwan Sriwattanapong, Thanakorn Theerapanon, Sermporn Thaweesapphithak, Wanna Chetruengchai, Thantrira Porntaveetus, Vorasuk Shotelersuk

**Affiliations:** ^1^Geriatric Dentistry and Special Patients Care International Program, Faculty of Dentistry, Chulalongkorn University, Bangkok, Thailand; ^2^Genomics and Precision Dentistry Research Unit, Department of Physiology, Faculty of Dentistry, Chulalongkorn University, Bangkok, Thailand; ^3^Center of Excellence for Regenerative Dentistry, Faculty of Dentistry, Chulalongkorn University, Bangkok, Thailand; ^4^Center of Excellence for Medical Genomics, Medical Genomics Cluster, Department of Pediatrics, Faculty of Medicine, Chulalongkorn University, Bangkok, Thailand; ^5^Excellence Center for Genomics and Precision Medicine, King Chulalongkorn Memorial Hospital, Thai Red Cross Society, Bangkok, Thailand

**Keywords:** hypodontia, oligodontia, nonsynonymous, homozygous, heterozygous, ectoderm

## Abstract

Tooth agenesis is one of the most common orodental anomalies that demonstrate phenotypic and genotypic heterogeneity with a prevalence of 2.5%–7%. Mutations in *WNT10A* have been proposed to be the most common cause of nonsyndromic tooth agenesis (NSTA). The aim of this study was to characterize the dental features and genetic variants of NSTA in a Thai population. We recruited 13 unrelated patients with NSTA who attended the Faculty of Dentistry, Chulalongkorn University, Thailand, from 2017 to 2019. All 13 underwent whole exome sequencing that identified likely pathogenic genetic variants, all in *WNT10A*, in five patients. All five patients had second premolar agenesis, while three also had absent or peg-shaped upper lateral incisors. Patient 1 possessed a novel heterozygous duplication, c.916_918dupAAC (p.Asn306dup) in *WNT10A.* Patients 2 and 3 harbored a heterozygous and homozygous c.637G > A (p.Gly213Ser) in *WNT10A*, respectively. Patients 4 possessed a heterozygous c.511C > T (p.Arg171Cys) in *WNT10A*. Patient 5 harbored a homozygous c.511C > T (p.Arg171Cys) in *WNT10A* and a novel heterozygous c.413A > T (p.Asn138Ile) in *EDARADD*, suggesting digenic inheritance. We recruited another 18 family members of these five patients. Out of 23 participants, homozygous *WNT10A* variants were identified in 2 patients and heterozygous variants in 17 individuals. Both homozygous patients had NSTA. Eight out of 17 heterozygous individuals (8/17) had NSTA or a peg-shaped lateral incisor, indicating a 47% penetrance of the heterozygous variants or 53% (10/19) penetrance of either homozygous or heterozygous variants in *WNT10A*. The frequencies of the c.511C > T in our in-house 1,876 Thai exome database, Asian populations, and non-Asian populations were 0.016, 0.005–0.033, and 0.001, respectively; while those of the c.637G > A were 0.016, 0.004–0.029, and 0.000, respectively. In conclusion, our study reports two novel variants with one each in *WNT10A* and *EDARADD*, expanding the genotypic spectra of NSTA. Second premolar agenesis is a common phenotype in affected individuals with variants in *WNT10A*; however, its penetrance is incomplete. Lastly, the different frequencies of *WNT10A* variants, c.511C > T and c.637G > A, in diverse populations might contribute to the prevalence range of NSTA between continents.

## Introduction

Tooth agenesis is the most common developmental dental anomaly in humans with a prevalence between 2.5 and 7% (Polder et al., 2004; Khalaf et al., 2014). Missing less than 6 teeth is called hypodontia, agenesis of 6 or more teeth is called oligodontia, and the complete absence of teeth is called anodontia. Multiple signaling pathways, including bone morphogenic protein, fibroblast growth factor, sonic hedgehog, and wingless (WNT), play important roles in the epithelial-mesenchymal interactions during tooth development. WNT/β-catenin signaling is involved from the early to late stages of tooth formation (Liu et al., 2008; Porntaveetus et al., 2012; Intarak et al., 2018). Among *WNT* family members, mutations in *WNT10A* (OMIM ^∗^606268) are predominantly related to tooth agenesis (TA) involving both nonsyndromic/isolated/selective tooth agenesis (NSTA/STHAG4, MIM #150400) and syndromic tooth agenesis, such as odontoonychodermal dysplasia (MIM #257980) and Schopf-Schulz-Passarge syndrome (MIM #224750).

Population-based studies have revealed that 28%–62% of tooth agenesis patients have with *WNT10A* variants (van den Boogaard et al., 2012; Mostowska et al., 2013; Arzoo et al., 2014). Heterozygous, homozygous, and compound heterozygous forms of *WNT10A* were associated with NSTA with phenotypic heterogeneity. Using *WNT10A* target sequencing, significantly elevated frequencies of *WNT10A* variants were observed in the tooth agenesis group compared with the control group (Song et al., 2014; Machida et al., 2017). Biallelic *WNT10A* variants were proposed to be a pathogenic factor for tooth agenesis with complete penetrance, while a single allelic variant, presenting in a significantly higher frequency in tooth agenesis patients, was considered to be a predisposing factor for tooth agenesis with reduced penetrance (Mues et al., 2014; Guazzarotti et al., 2018). These findings prompted us to investigate the dental phenotype and genotype in Thai patients with NSTA and determine the allele frequencies of *WNT10A* in Thais compared with Asian and non-Asian populations.

## Materials and Methods

### Subject Enrollment

The study protocol was approved by the Institutional Review Board (HREC-DCU 2018-091), Faculty of Dentistry, Chulalongkorn University and complied with the Declaration of Helsinki. Written informed consents were obtained prior to the patients’ participation in this study. Thirteen unrelated patients with NSTA who attended the Faculty of Dentistry, Chulalongkorn University, Thailand between January 2017 and March 2019 and their family member were recruited. Clinical and radiographic examinations, dental history, and intraoral photographs of the probands were used to assess tooth agenesis. The size and shape of the remaining teeth were also observed. The patients did not have any signs or symptoms related to ectodermal organ defects, e.g. intolerance to heat, dry skin, abnormal sweating, sparse hair, or brittle nails (Bergendal et al., 2006). The dental phenotypes of the probands’ family members were obtained from clinical and radiographic examinations, dental records, or participant interviews.

### Mutation Analyses

Genomic DNA extracted from peripheral blood leukocytes was subjected to mutation analysis using whole exome sequencing (WES) (Porntaveetus et al., 2018). Briefly, genomic DNA was captured using a SureSelect Human All Exon version 4 kit (Agilent Technologies, Santa Clara, CA, United States) and sequenced using Hiseq2000 (Macrogen, Seoul, South Korea). The sequences were aligned to the human genome reference sequence^1^ using the Burrows-Wheeler Aligner^2^. Downstream processing was performed with SAMtools^3^ and annotated against the dbSNP and 1000 Genomes. After quality filtering, the variants were screened using the genes listed in HP: 0009804 (reduced number of teeth) in Human Phenotype Ontology (Köhler et al., 2018). All calls with a coverage < 10×, minor allele frequency > 5% in the 1000 Genomes Project, Genome Aggregation Database (gnomAD^4^), and our in-house database of 1,876 Thai exomes; non-coding variants; and synonymous exonic variants were filtered out. The identified variants were confirmed by Sanger sequencing (Supplementary Table 1). The alignment and conservation of amino acid residues were generated by Clustal Omega (Madeira et al., 2019).

Allele frequencies of *WNT10A* variants were screened with multiple variant databases comprising the Genome Aggregation Database (gnomAD), GenomeAsia100K consortium, Northeast Asian Reference Database (NARD), Han Chinese genome project (PGG.Han), 4.7K Japanese individual genome variation (4.7KJPN), Human Genetic Variation Database (HGVD), Korean Variant Archive (KOVA), and our in-house database, last access on June 3, 2020.

Bioinformatics tools consisting of PolyPhen-2 (Adzhubei et al., 2010), SIFT (Kumar et al., 2009), MutationTaster (Schwarz et al., 2014), and CADD (Rentzsch et al., 2018) were used to predict each variant’s pathogenicity.

## Results

**FIGURE 1:**
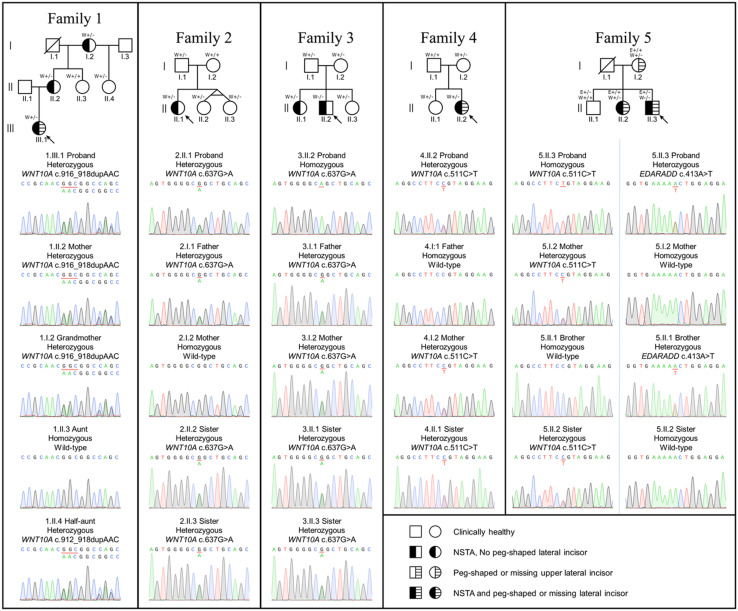
Family pedigrees and genetic variants. Arrow indicates the proband. Tooth phenotypes of the participants were determined either by the dentist or reported by the participants. W+/+, homozygous *WNT10A* variant; W+/-, heterozygous *WNT10A* variant; W+/+, wild-type *WNT10A* variant; E+/+, homozygous *EDARADD* variant; E+/-, heterozygous *EDARADD* variant; E+/+, wild-type *EDARADD* variant.

We performed WES for 13 unrelated patients with NSTA during 2017–2019 and detected variants related to tooth agenesis (HP: 0009804) in five patients. All five patients (Patients 1–5) possessed variants in *WNT10A* (NM_025216.3). We then recruited 18 additional family members of these 5 index patients, characterized their dental phenotype, and performed Sanger sequencing (Figure 1 and Supplementary Figure 1).

Patient 1, a 16-year-old female, lacked nine permanent teeth, all premolars and the lower right first molar. The lower left second molar was extracted due to pulp necrosis. Oral examination revealed that she had peg-shaped upper lateral incisors, severely hypoplastic edentulous ridges, anterior deep bite, and malocclusion. WES identified that the patient possessed a novel heterozygous duplication, c.916_918dupAAC (p.Asn306dup), in *WNT10A* (ClinVar SCV001335264). This variant was detected in the patient’s mother and grandmother who had NSTA, and in the unaffected half aunt.

Patient 2, a 27-year-old female, had 6 missing teeth, 4 upper premolars, and 2 lower third molars. The known heterozygous missense mutation, c.637G > A (p.Gly213Ser), in *WNT10A* was identified in the patient, and in the patient’s father and two sisters who reported no missing teeth. The mothers had biallelic wild-type alleles.

Patient 3, a 28-year-old male, had 12 missing teeth comprising an upper right canine, 3 first premolars, 4 second premolars, and 4 third molars. The homozygous mutation, c.637G > A (p.Gly213Ser), in *WNT10A* was identified in the patient. The *WNT10A* heterozygous c.637G > A (p.Gly213Ser) variant was detected in the patient’s older sister who was missing her 4 second premolars, and the younger sister, father, and mother who did not have any teeth missing.

Patient 4, a 31-year-old female, was missing her 2 lower second premolars and peg-shaped upper lateral incisors. Her deciduous lower right and left second molars were retained. Four third molars were previously extracted. The known heterozygous missense variant, c.511C > T (p.Arg171Cys), in *WNT10A* was detected in the patient, and in the patient’s older sister and mother who did not have tooth agenesis. The father possessed wild-type alleles.

Patient 5, a 34-year-old male, had agenesis of 8 permanent teeth, 2 upper lateral incisors, 2 right second premolars, and 4 third molars. He had implant replacements at the upper right lateral incisor and lower right second premolar. The homozygous missense variant, c.511C > T (p.Arg171Cys) in *WNT10A* and a novel heterozygous missense variant, c.413A > T (p.Asn138Ile), in *EDARADD* (Clinvar SCV001335265) was identified in the patient. The heterozygous *WNT10A* c.511C > T variant was also detected in the patient’s older sister who was missing two upper lateral incisors and two lower third molars and the mother who had peg-shaped upper lateral incisors. The heterozygous *EDARADD* c.413A > T variant was present in the patient’s older brother, but not in the sister and mother.

In Patients 1–5, the number of missing teeth was between 2 and 12 or between 2 and 9 teeth (excluding the third molars). An absent second premolar or peg-shaped upper lateral incisor was frequently observed in the patients (Table 1).

**TABLE 1:** The identified *WNT10A* variants in subjects with tooth agenesis or peg-shaped upper lateral incisors.

**Family**	**Patient**	**Dental arch**	**Tooth**																			
			**8**	**7**	**6**	**5**	**4**	**3**	**2**	**1**	**1**	**2**	**3**	**4**	**5**	**6**	**7**	**8**	**Number of missing teeth (excluding 3^rd^ molars)**	**Mode**	**Mutation**	**Amino acid change**
1	Patient 1	Upper				**•**	**•**		□			□		**•**	**•**							
		Lower			**•**	**•**	**•**							**•**	**•**		**x**		9	Het	c.916_918dup	p.Asn360dup
2	Patient 2	Upper				**•**	**•**							**•**	**•**							
		Lower	**•**															**•**	6 (4)	Het	c.637G > A	p.Gly213Ser
3	Patient 3	Upper	**•**			**•**	**•**	**•**						**•**	**•**			**•**				
		Lower	**•**			**•**								**•**	**•**			**•**	12 (8)	Homo	c.637G > A	p.Gly213Ser
	Patient 3’s older sister	Upper				**•**									**•**							
		Lower	**□**			**□**								**□**	**□**			**□**	2	Het	c.637G > A	p.Gly213Ser
4	Patient 4	Upper							□			□										
		Lower				**•**									**•**				2	Het	c.511C > T	p.Arg171Cys
5	Patient 5^a^	Upper	**•**			**•**			■			■						**•**				
	(II.3)	Lower	**•**			**•**												**•**	8 (4)	Homo	c.511C > T	p.Arg171Cys
	Patient 5’s sister	Upper							■			■										
	(II.2)	Lower	■			**□**												**•**	4 (2)	Het	c.511C > T	p.Arg171Cys
	Patient 5’s mother	Upper							□			□										
	(I.2)	Lower																	0	Het	c.511C > T	p.Arg171Cys

*1, central incisor; 2, lateral incisor; 3, canine; 4, first premolar; 5, second premolar; 6, first molar; 7, second molar; 8, third molar.; •, missing tooth; ■, missing upper lateral incisor; □, peg-shaped upper lateral incisors; x, extraction; NA, not available; Het, heterozygous; Homo, homozygous.^*a*^The heterozygous missense variant, c.413A > T (p.Asn138Ile), in *EDARADD* was also found in Patient 5.*

We used several bioinformatic tools to predict the pathogenicity of the variants. We found that the *WNT10A* c.511C > T (p.Arg171Cys) was predicted to be deleterious (CADD: 25.6), deleterious (SIFT: 0.0), possibly damaging (PolyPhen-2: 0.93), and disease causing (MutationTaster). The *WNT10A* c.637G > A (p.Gly213Ser) was predicted to be deleterious (CADD: 27.2), deleterious (SIFT: 0.0), probably damaging (PolyPhen-2: 1.0), and disease causing (MutationTaster). Furthermore, the *EDARADD* c.413A > T (p.Asn138Ile) was predicted to be deleterious (CADD: 27.3), deleterious (SIFT: 0.0), possibly damaging (PolyPhen: 1.0), and disease causing (MutationTaster).

The amino acid residues; p.Arg171, p.Gly213, and p.Asn306 in WNT10A, and p.Asn128 in EDARADD are conserved among multiple species (Supplementary Figure 2). According to the ACMG standards and guidelines, the *WNT10A* c.511C > T and c.637G > A and the *EDARADD* c.413A > T variants are considered to be likely pathogenic, while the *WNT10A* c.916_918dupAAC variant is considered as uncertain significance (Richards et al., 2015).

Eighteen members of the five index patients’ families (23 total) were included. Out of the 23 participants, the homozygous *WNT10A* variants were identified in 2 patients (3.II.2 and 5.II.3) and the heterozygous variants in 17 individuals. Both homozygous patients had nonsyndromic tooth agenesis (NSTA). Eight (1.I.2, 1.II.2, 1.III.1, 2.II.1, 3.II.1, 4.II.2, 5.I.2, and 5.II.2) out of 17 heterozygous individuals (8/17) had NSTA or a peg-shaped lateral incisor, indicating a 47% penetrance of the heterozygous variants, or a 53% (10/19) penetrance of either homozygous or heterozygous variants in *WNT10A.*

We screened the frequencies of the *WNT10A* c.511C > T and c.637G > A variants in multiple variant databases comprising the Genome Aggregation Database (gnomAD), GenomeAsia100K consortium, Northeast Asian Reference Database (NARD), Han Chinese genome project (PGG.Han), 4.7K Japanese individual genome variation (4.7KJPN), Human Genetic Variation Database (HGVD), Korean Variant Archive (KOVA), and our in-house database. The allele frequencies of *WNT10A* c.511C > T and c.637G > A in Asian populations was between 0.005 and 0.033 and between 0.004 and 0.029, respectively. In our in-house database (ThWES) of 1,876 Thai exomes, the frequency of c.511C > T variant was 0.016 and that of c.637G > A variant was 0.016, which were in the Asian population ranges. In contrast, the frequency of the c.511C > T variant was 0.001 and that of the c.637G > A variant was and 0.000 in non-Asian populations (Table 2). These results indicate that the WNT10A c.511C > T and c.637G > A variants are common and concentrated in Asian populations compared with non-Asian populations.

**TABLE 2:** Allele frequencies and details of *WNT10A* c.511C > T (p.Arg171Cys) and c.637G > A (p.Gly213Ser) variants.

	**rs116998555 c.511C > T (p.Arg171Cys)**				**rs147680216 c.637G > A (p.Gly213Ser)**			
**Database**	**Allele count**	**Allele number**	**Number of homozygotes**	**Allele frequency**	**Allele count**	**Allele number**	**Number of homozygotes**	**Allele frequency**
**gnomAD (v2.1.1 and v3)**								
East Asian	370	23084	3	0.016	657	22960	16	**0.029**
Non-East Asian	335	402864	1	0.001	22	400838	0	0.000
*South Asian*	51	33652	1	0.002	4	33616	0	0.000
*African*	32	67004	0	0.001	3	66550	0	0.000
*Ashkenazi Jewish*	0	13692	0	0.000	0	13646	0	0.000
*European (Finnish)*	13	35558	0	0.000	0	35046	0	0.000
*European (non-Finnish)*	223	193594	0	0.001	4	192768	0	0.000
*Latino*	6	49094	0	0.000	3	48994	0	0.000
*Other*	10	10270	0	0.001	8	10218	0	0.001
Total	705	425948	4	0.002	679	423798	16	0.002
**GenomeAsia 100K**								
Southeast Asia	4	692	0	0.006	3	692	0	**0.004**
Northeast Asia	5	702	0	0.007	10	702	0	0.014
South Asia	7	1448	0	**0.005**	0	1448	0	0.000
Other	0	636	0	0.000	0	636	0	0.000
Total	16	3478	0	0.005	13	3478	0	0.004
Northeast Asian Reference Database (NARD)	35	3558	NA	0.010	51	3558	NA	0.014
Han Chinese genome project (PGG.Han)	3596	107232	NA	**0.033**	1161	108146	NA	0.011
4.7K JPN (4,773 Japanese individuals)	NA	NA	NA	0.010	NA	NA	NA	0.015
HGVD (3,248 Japanese individuals)	36	2412	0	0.015	35	2388	0	0.015
Korean Variant Archive (KOVA) (1,055 healthy Korean individuals)	NA	NA	NA	0.019	NA	NA	NA	0.019
ThWES 1876 (In-house database of 1,876 Thai individuals)	58	3752	4	0.016	59	3752	0	0.016

*JPN, Japanese individual genome variation; genomAD, Genome Aggregation Database; HGVD, Human Genetic Variation Database; NA, not available. Bold numbers indicate maximum and minimum allele frequencies in the column.*

## Discussion

In this study, we identified five index patients having NSTA and variants in *WNT10A*. Eighteen more family members were included. The number of missing teeth observed ranged from 2 to 12 teeth or 2 to 9 teeth, excluding third molars. *WNT10A* is the most common variant associated with NSTA (van den Boogaard et al., 2012; Mostowska et al., 2013; Arzoo et al., 2014). Here, we identified that all five patients possessed *WNT10A* variants. The novel heterozygous duplication, c.916_918dupAAC (p.Asn306dup), was identified in Patient 1. The heterozygous state of c.637G > A (p.Gly213Ser) variant was detected in Patient 2 and its homozygous state in Patient 3. The heterozygous state of c.511C > T (p.Arg171Cys) variant was detected in Patient 4 and its homozygous state was detected in Patient 5. The novel heterozygous c.413A > T (p.Asn138Ile) in *EDARADD* was observed in Patient 5.

The relationship between the heterozygous *WNT10A* variants (c.511C > T and c.637G > A) and NSTA was characterized in large well-phenotyped population cohorts (Song et al., 2014; Machida et al., 2017). Both variants were significantly associated with tooth agenesis compared with healthy control individuals. In other studies, the variants were shown to cause tooth agenesis with incomplete penetrance (He et al., 2013; Plaisancié et al., 2013; Kantaputra et al., 2014). Biallelic *WNT10A* variants were proposed to be the pathogenic factor for tooth agenesis with complete penetrance, while single allelic variants, presenting with a significantly higher frequency in tooth agenesis patients, were considered to be a predisposing factor for tooth agenesis with reduced penetrance (Mues et al., 2014; Guazzarotti et al., 2018). In addition, the phenotypic spectrum of *WNT10A* mutations was shown to be dose-dependent with variable expressivity, including within the same family (Park et al., 2019). Patients with biallelic *WNT10A* mutations had severe tooth agenesis, while heterozygous patients were either unaffected or had a mild tooth phenotype.

In our study, Patient 3 (3.II.2) and Patient 5 (5.II.3), who harbor the homozygous *WNT10A* c.637G > A and c.511C > T variants, respectively, have tooth agenesis and their phenotypes are more severe than the heterozygous individuals with the same variant. In our cohort, the heterozygous c.511C > T and c.637G > A variants demonstrated incomplete penetrance, which is consistent with previous reports. The evidence mentioned above suggests that the heterozygous *WNT10A* c.511C > T or c.637G > A allele can be a contributing factor for NSTA with low penetrance, while biallelic variants are associated with greater clinical severity. However, there might be other genetic or environmental factors influencing the phenotypic expression of NSTA patients.

Mutations in *EDARADD* (OMIM^∗^ 606603) are associated with autosomal dominant ectodermal dysplasia 11A (MIM# 614940) (Bal et al., 2007; Cluzeau et al., 2011) and autosomal recessive ectodermal dysplasia 11B (MIM# 614941) (Chaudhary et al., 2016). The *EDARADD* c.413A > T (p.Asn138Ile) variant identified in Patient 5 is located in the death domain that interacts with EDAR. The homozygous and heterozygous mutations in the EDAR death domain cause hypohidrotic ectodermal dysplasia (MIM #614940, #614941) with low penetrance (Bal et al., 2007; Cluzeau et al., 2011). To the best of our knowledge, only one heterozygous *EDARADD* variant, c.308C > T, p.Ser103Phe, was identified in patients with NSTA. The allelic frequency of the *EDARRADD* c.308C > T variant is found in up to 2% of a healthy population according to the dbSNP database. However, this variant has been associated with NSTA, but with low penetrance and variable expressivity. (Bergendal et al., 2011; Arte et al., 2013; Barbato et al., 2018; Martínez-Romero et al., 2019). Although the heterozygous *EDARADD* c.413A > T mutation was detected in the patient and his unaffected brother, it was not in his affected sister or mother.

Interestingly, the digenic heterozygous variants of *WNT10A* and other genes in the EDA pathway, including *EDA, EDAR*, and *EDARADD*, have been found in several patients with NSTA (Arte et al., 2013; He et al., 2013; Barbato et al., 2018; Martínez-Romero et al., 2019). The WNT and EDA pathways are suggested to play complementary roles during tooth development (Yu et al., 2019). In Family 5, the *WNT10A* c.511C > T variant was present in the homozygous state in the patient (8 missing teeth), in the heterozygous state in the sister (4 missing teeth) and proband’s mother (peg-shaped lateral incisors), and not detected in the proband’s brother (unaffected). Therefore, the role of the *WNT10A* variant in this family may be because its homozygous state is associated with more severe NSTA than those with the heterozygous variant, and the heterozygous variant shows incomplete penetrance. Moreover, the coexistence and variable penetrance of *WNT10A* and *EDARADD* variants may modulate the final phenotype of Patient 5.

Tooth agenesis is one of the most common anomalies in human development. Its prevalence in the general population is 2.5–7% (Polder et al., 2004). Mutations in *WNT10A* are the most frequently found variants associated with NSTA in several populations studied to date (van den Boogaard et al., 2012; Mostowska et al., 2013; Song et al., 2014; Tardieu et al., 2017). In particular, the *WNT10A* c.511C > T and c.637G > A variants are predominant in Asian populations compared with Europeans (Song et al., 2014; Machida et al., 2017). According to multiple genetic databases, we observed the allele frequencies of *WNT10A* c.511C > T and c.637G > A variants in Asian populations up to 0.033 and 0.029, respectively, compared with those in non-Asians, which are 0.000–0.001. The frequencies of the c.511C > T and c.637G > A variants in our in-house database of 1,876 Thai exomes are 0.016, which are in the range of Asian populations, including Japanese, Chinese, and Koreans. These results indicate that these two variants are relatively common in Asian populations. The difference in *WNT10A* allele frequencies among different ethnic groups may also partly explain the diverse prevalence of tooth agenesis on different continents (Khalaf et al., 2014).

The number and location of missing teeth are associated with mutations in specific genes (Al-Ani et al., 2017). A genotype-phenotype correlation study revealed that the second premolars were the most common missing teeth found in patient with *WNT10A* variants (Arzoo et al., 2014). All five patients in our study had agenesis of the second premolars, suggesting that *WNT10A* variants might be responsible for the absence of second premolars with high penetrance. Mutations in *WNT10A* have also been proposed to cause agenesis or microdontia of the upper lateral incisors (Kantaputra et al., 2014; Mostowska et al., 2015). Absent upper lateral incisors was observed in Patient 5 and his sister, and peg-shaped upper lateral incisors were found in Patient 1, Patient 4, and Patient 5’s mother who had the heterozygous *WNT10A* variants.

## Conclusion

In conclusion, this study reports a novel in-frame duplication, c.916_918dupAAC (p.Asn306dup), in *WNT10A* and a novel heterozygous missense variant, c.413A > T (p.Asn138Ile), in *EDARADD*, expanding the genotypic spectrum related to NSTA. The heterozygous *WNT10A* c.511C > T and c.637G > A variants demonstrate incomplete penetrance. Both variants are more common in Asian populations compared with non-Asians, which might explain the diverse prevalence of NSTA in various continents.

## Data Availability Statement

The datasets presented in this study can be found in online repositories. The names of the repository/repositories and accession number(s) can be found in the article/Supplementary Material.

## Ethics Statement

The studies involving human participants were reviewed and approved by the Institutional Review Board (HREC-DCU 2018-091), Faculty of Dentistry, Chulalongkorn University and were complied with the Declaration of Helsinki. Written informed consent to participate in this study was provided by the participants’ legal guardian/next of kin. Written informed consent was also obtained from the individual(s), and minor(s)’ legal guardian/next of kin, for the publication of any potentially identifiable images or data included in this article.

## Author Contributions

CK and TT contributed to the data acquisition and drafting of the manuscript. KS, ST, and WC contributed to the analysis and interpretation of the results. TP contributed to the study conception and design and drafting of the manuscript. VS contributed to the conception and data analysis. All authors revised and approved the submitted version.

## Conflict of Interest

The authors declare that the research was conducted in the absence of any commercial or financial relationships that could be construed as a potential conflict of interest. The reviewer MA declared a past co-authorship with the authors TT and TP to the handling editor.

## Footnotes

1 UCSC Genome Browser, hg19

2 http://bio-bwa.sourceforge.net/

3 samtools.sourceforge.net/

4 gnomad.broadinstitute.org

## Supplementary Material

Supplementary Figure 1 Clinical and radiographic features of the patients. ^∗^indicates missing tooth.

Supplementary Figure 2 Schematic diagrams of the *WNT10A* and *EDARADD* genes and proteins showing the identified *WNT10A* variants c.916_918dupAAC (p.Asn306dup), c.637G > A (p.Gly213Ser), and c.511C > T (p.Arg171Cys).

Supplementary Table 1 Primers for Sanger sequencing.
