# Supplementary material for: Phenotypic and Genotypic Features of Thai Patients With Nonsyndromic Tooth Agenesis and WNT10A Variants
Source: Front Physiol. 2020 Nov 19;11:573214. doi: 10.3389/fphys.2020.573214 (PMC7710930; doi:10.3389/fphys.2020.573214)
Supplement: Supplementary Figure 1 — Clinical and radiographic features of the patients. ∗indicates missing tooth. [file Data_Sheet_1.PDF]

Supplementary Material

Supplementary Table 1. Primers for Sanger sequencing.

Gene	Transcript	Mutation	Exon	Sequences
WNT10A	NM_025216.3	c.916_918dupAAC (p.Asn306dup)	4	F: AGTGGGTTTCAGAAGCAGGC
				R: GAAGTAGACCAGGTCGGCG
WNT10A	NM_025216.3	c.511C>T (p.Arg171Cys), c.637G>A (p.Gly213Ser)	3	F: ACTGGCTTCTGGCGTGATTT
				R: TCCCGGGAGTCCAGAAAGTC
EDARADD	NM_145861.4	c.413A>T (p.Asn138Ile)	6	F: GCCTCTTGTTGACCTGTGGA
				R: AACATCCGGAGAGGCCAATG

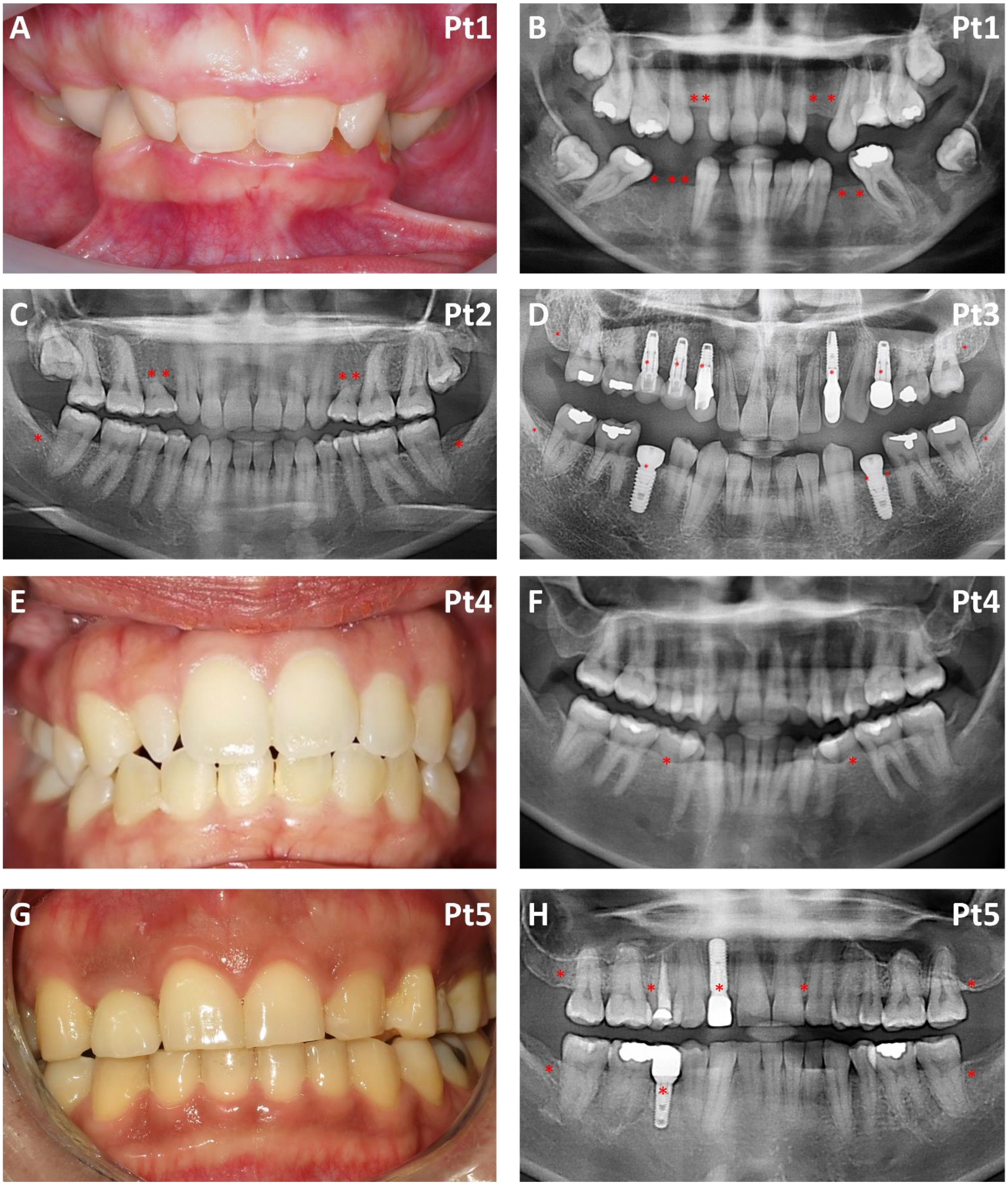

Supplementary Figure 1. Clinical and radiographic features of the patients. * indicates missing tooth.

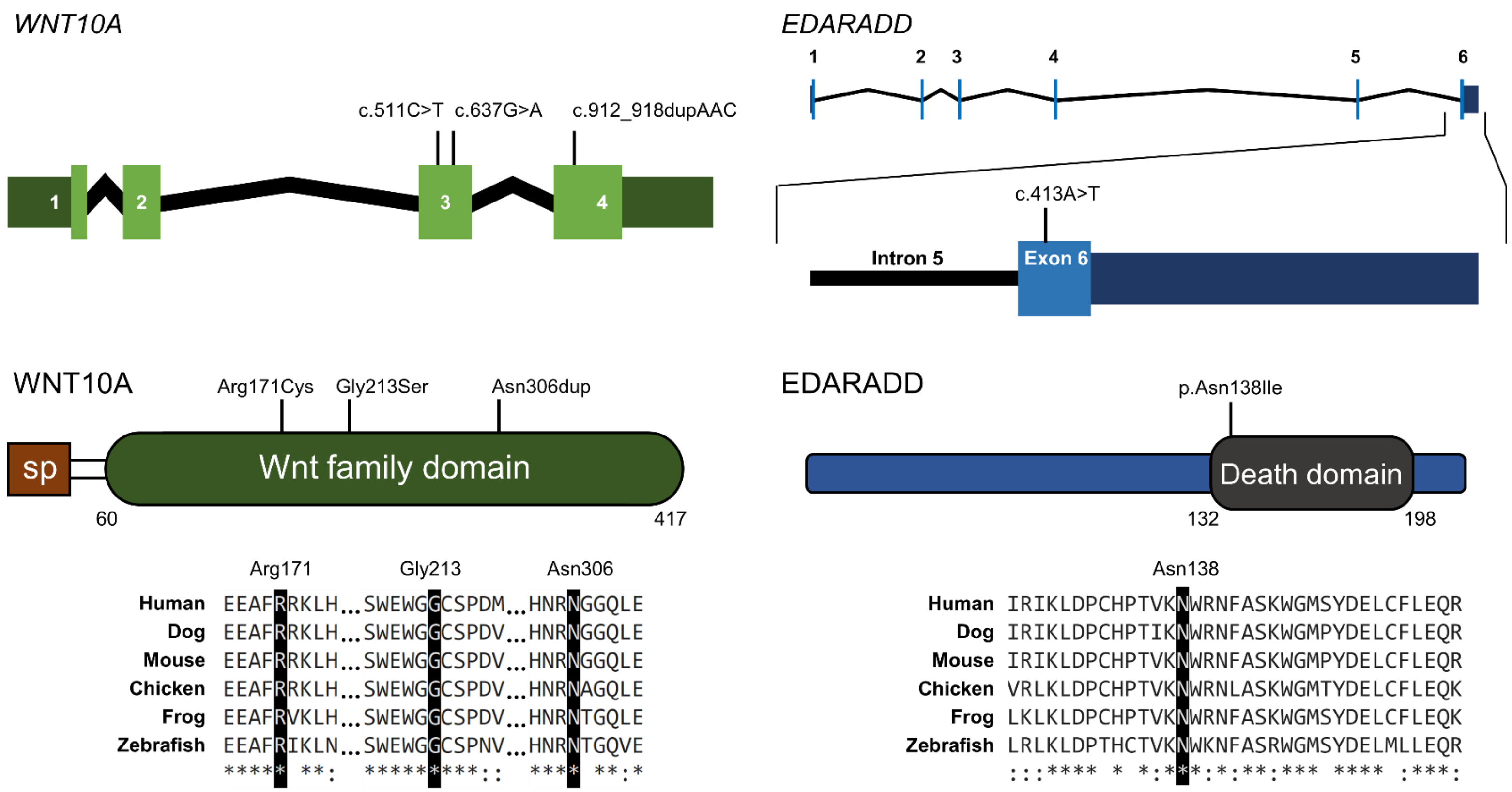

Supplementary Figure 2. Schematic diagrams of *WNT10A* and *EDARADD* genes and proteins showing the identified *WNT10A* variants c.916_918dupAAC (p.Asn306dup), c.637G>A (p.Gly213Ser), and c.511C>T (p.Arg171Cys).
